# Prevalence and Associated Factors of Excessive Dietary Supplement Use Among Japanese Adults: Cross-Sectional Study

**DOI:** 10.2196/82623

**Published:** 2026-03-19

**Authors:** Minami Sugimoto, Keiko Asakura, Nana Shinozaki, Kentaro Murakami

**Affiliations:** 1 Department of Environmental and Occupational Health, School of Medicine, Toho University, Tokyo, Japan; 2 Department of Preventive Medicine, School of Medicine, Toho University, Omori-Nishi 5-21-16, Ota-ku, Tokyo, 143-8540, Japan, 81 3-3762-4151, 81 3-5493-5416; 3 Department of Social and Preventive Epidemiology, School of Public Health, The University of Tokyo, Tokyo, Japan

**Keywords:** dietary supplement, Japanese, risk factors, nutrients, dietary reference intake

## Abstract

**Background:**

The use of dietary supplements (DSs) can lead to an excessive intake of certain nutrients, posing potential health risks. However, studies are scarce on whether DS users adhere to recommended doses provided by the manufacturer and what factors are associated with overconsumption of DS.

**Objective:**

By leveraging purchase history data to estimate DS use precisely, this study aimed to investigate the factors associated with DS consumption exceeding manufacturer-recommended doses and examine the prevalence of excess nutrient intake among DS users.

**Methods:**

An online survey was conducted from November to December 2024 among 2002 adults (aged 18‐74 years) with a history of buying one of the 25 major DS products and who had used it in the previous month or regularly. Self-reported daily DS consumption was assessed using a questionnaire and compared to the manufacturer-recommended doses indicated in the package. Using multivariate logistic regression analysis, the associations between DS consumption exceeding the manufacturer-recommended doses and sociodemographic factors were examined.

**Results:**

The prevalence of DS users exceeding tolerable upper intake levels (ULs), defined according to the Dietary Reference Intakes for Japanese, was calculated based on vitamin and mineral intake from DS only. Consequently, 371 (18.5%) of the 2002 participants consumed DS above the manufacturer-recommended dose. Consumption of DS above the recommended dose was associated with middle-aged, part-time or full-time employment, use of tablet-form DS, especially single water-soluble vitamin tablets, use of DS for 6 months or more, and intentional consumption above the recommended dose. For 1705 individuals consuming DS with UL nutrients, 17.4% (n=297) exceeded the manufacturer-recommended dose. Of these, 61.9% (184/297) surpassed UL by ≥1 nutrient.

**Conclusions:**

Middle-aged, part-time or full-time jobs, using tablet-form DS, and using DS for 6 months or more were associated with DS consumption exceeding the manufacturer-recommended dose, which may lead to excessive nutrient intake.

## Introduction

Over the last few decades, the use of dietary supplements (DSs) has been increasing in many countries [1-4]. For example, the proportion of DS users in the United States increased from 51.8% in 2011‐2012 to 61.4% in 2021‐2023 [3], while in Australia, it increased from 28.5% in 2011‐2012 to 33.6% in 2023 [4]. Although DSs are widely used to promote health and prevent nutrient deficiencies, concerns have emerged regarding their safety due to DS overuse and the risk of excessive nutrient intake [5,6]. Since primary sources of vitamins and minerals are a normal diet [7], having nutrients from DS in addition to diet might increase the risk of exceeding the tolerable upper intake level (UL). Excessive nutrient intake could bring adverse health effects such as liver dysfunction by vitamin A and hypercalcemia by vitamin D and calcium [8]. Therefore, understanding the behaviors and factors that contribute to DS overuse and excessive nutrient intake is a pressing public health issue.

Previous studies have investigated the prevalence [6,7,9-15] and risk factors [16] of exceeding the ULs when considering DS consumption. For instance, approximately 10% of DS users surpass the UL for iron or zinc, and <5% exceed the UL for calcium or magnesium among adults in the United States [9]. In Ireland, the risk of excess was most notable for iron and vitamin B_6_ among women taking DS [12]. Although high-dose DS (ie, containing higher content of nutrients in one tablet or capsule) itself constitutes a risk for excessive nutrient intake [17], one possible cause of excessive nutrient intake among DS users is the overconsumption of DS products. In the United States and European countries, regulations mandate that DS product labels display the manufacturer-recommended dose (written as “serving size” or “recommended daily intake”) to safeguard consumers [18,19]. However, studies on whether DS users adhere to manufacturer-recommended doses are scarce. For example, a 2012 survey by the Consumer Commission, Cabinet Office, Japan, reported that 2.4% of 8064 DS users consumed DS exceeding doses recommended by the manufacturer [20]. The prevalence of adherence to manufacturer-recommended doses may have been underestimated because the survey relied on self-reports to assess adherence to DS use, namely asking participants whether their consumption aligned with, below, or above the recommended dose.

Most previous studies [2-4,7,9-16], as well as the aforementioned Japanese survey [20], relied on self-reported data to collect information on the consumption of DS products and their use. This approach has several limitations. Participants may misreport or forget the details regarding DS use, making it challenging to identify the exact products used [21]. Furthermore, DS databases have rapidly become outdated because of the rapid turnover of products in the market [21]. These methodological weaknesses hinder the accurate estimation of DS consumption and compliance. To address these issues, purchase history data can be useful for precisely identifying DS products. Using purchase history data enables us to ask about the usage habits of specific products among participants without self-reporting the product names. This prevents the generation of data that is unanalyzed due to unknown product names. By leveraging purchase history data, this study aimed to address these gaps, with the following objectives: (1) to investigate the factors associated with DS consumption exceeding the manufacturer-recommended doses indicated on the product label or manufacturer website and (2) to examine the prevalence of nutrient intake exceeding the tolerable upper limits among DS users. This study provides a methodologically robust alternative to traditional self-reporting approaches. Since no previous study has investigated factors associated with overuse of DS, we analyzed, as a first step, variables related to sociodemographic and socioeconomic status and dietary supplement use habits.

## Methods

### Participants and Overview of Study Design

This cross-sectional study used data obtained through a web-based questionnaire survey. Data were collected between November 14 and December 1, 2024. This study was prepared according to the Checklist for Reporting Results of Internet e-Surveys (CHERRIES) [22]. The process of participant selection included identifying commonly used DS and subsequently selecting participants who reported using them. The survey procedure is detailed in the following sections. Target participants were Japanese adults (≥18 years) who consumed at least one DS product in the previous month. The target sample size was set at 2000 adults. This sample size allowed for reasonably precise estimates of the prevalence of nutrient intake exceeding the ULs, while also remaining feasible within financial constraints.

Survey participants were recruited from a registered consumer panel. The panelists routinely recorded their purchased products (such as food, daily necessities, and DSs) through a dedicated smartphone app. This consumer panel comprised primary shoppers in each household, with 70% being females and 75% being in their 20s to 40s. The panelists were distributed across Japan, closely reflecting the national population distribution.

Although the consumer panel was managed by Research and Innovation Co. Ltd., the data collection process (including participant invitation, survey distribution, and data management) was conducted using the research infrastructure of INTAGE Inc., a major market research company in Japan and the parent company of Research and Innovation Co. Ltd. This was because Research and Innovation Co. Ltd. did not have a system for asking users detailed questions, whereas INTAGE Inc. did.

### Ethical Considerations

This study was approved by the ethics committee of the Toho University Faculty of Medicine (approval no. A24059; approved on October 7, 2024). Informed consent was obtained from all the participants on the website. To ensure privacy and confidentiality, the research team received only anonymized data from the survey company (INTAGE Inc.), and no personally identifiable information was accessible to the researchers. All data were handled in accordance with institutional ethical guidelines. The participants were compensated with standard incentives from Research and Innovation Co. Ltd, after completing the survey.

### Survey Procedure

#### Overview

**Figure 1.**
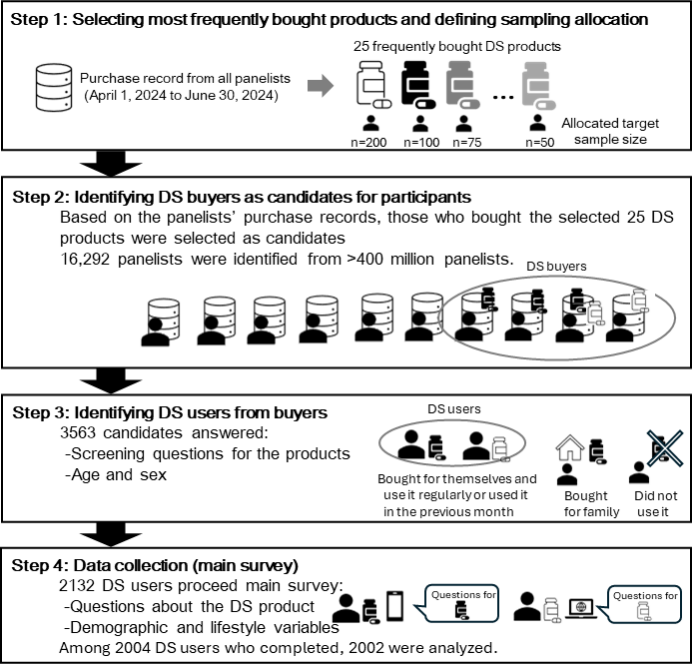
Survey procedure. DS: dietary supplement.

Data were collected in 4 steps (Figure 1). The first and second authors (MS and KA) prepared the questionnaires. Each participant reported information about one of the selected DS products.

#### Step 1: Selecting the Most Frequently Bought Products and Defining Sampling Allocation

Due to the lack of comprehensive and nationwide statistical data on the consumption of DS products, the most frequently purchased DS products are unknown. Thus, using purchase record data from all panelists for the past 3 months (April 1, 2024, to June 30, 2024), the second author (KA) identified the most frequently purchased DS products across the following 5 categories: liquid form supplements, multivitamin tablets, water-soluble vitamin tablets, fat-soluble vitamin tablets, and mineral tablets. The anticipated number of respondents was first allocated to each of the 5 categories to ensure accurate estimation of the prevalence of excessive intake. Considering the actual sales volumes, the anticipated number of respondents was allocated as follows: 300 for liquid-form DSs, 400 for multivitamin tablets, 600 for water-soluble vitamin tablets, 200 for fat-soluble vitamin tablets, and 500 for mineral tablets. To meet the target sample sizes, products were selected in descending order of sales volume within each category. The number of products selected in each category was as follows: 2 liquid-form DS, 6 multivitamin tablets, 6 water-soluble vitamin tablets, 7 fat-soluble vitamin tablets, and 4 mineral tablets, totaling 25 products. The names of the selected 25 DS products and the expected number of respondents for each product are presented in Table S1 in Multimedia Appendix 1.

#### Step 2: Identifying DS Buyers as Candidates for Participants

The buyers of the 25 DS products selected in step 1 were identified from over 4 million panelists by INTAGE Inc as candidates for participants. Panelists were selected as buyers of DS if they had at least one purchase history of ≥1 DS among 25 selected DS products in the past 3 months. To avoid duplicate responses from individuals, those with a record of purchasing 2 or more selected DS products were randomly assigned as potential respondents to one of the selected DS products. INTAGE Inc. operates a random allocation. Consequently, 16,292 panelists were identified as DS buyers (ie, candidates for participation).

#### Step 3: Identifying DS Users From Buyers (Screening)

INTAGE Inc. sent invitation emails to candidates. Participants completed the questionnaire via a survey platform provided by INTAGE Inc using their own smartphone, tablet, or PC. Through identity authentication, access to the questionnaire was restricted to the individual panelist surveyed. During the survey, the collected data were encrypted and stored in a database at INTAGE Inc. To screen DS users, the candidates were asked the following questions after a question about their sex and age:

- Question 1: We would like to ask you about the dietary supplement [product name] you have purchased in the past 3 months. Did you purchase this product for your use? (options: yes, for myself; no, for my family)
- Question 2: Do you still use this product regularly? (options: yes or no)
- Question 3: Have you taken this product in the last month? (options: yes or no)

Those who answered “yes” to question 1 and also responded “yes” to either or both questions 2 and 3 were identified as DS users. Among the 16,292 candidates, 3563 (21.9%) panelists answered the screening questions. After screening the questions, 1431 panelists were excluded, and 2132 (13.1%) of the 16,292 panelists were identified as DS users and proceeded to the main survey.

#### Step 4: Data Collection (Main Survey)

A total of 2132 participants were asked about the use of one of the 25 selected DS products. Among them, 106 participants did not complete the questionnaire, and 22 panelists were excluded because of unreliable data and an extremely short response time. Overall, 2004 participants completed the main survey at a completion rate of 94% (2004/2132). The main survey comprised 50 questions. The number of items per page was set to one to increase readability on the screen. Thus, the survey consisted of 50 screens in total.

### Assessment of DS Use

The participants were asked to respond to a series of questions regarding one of the 25 DS products purchased. To simplify the survey procedure, we did not ask questions about the use of other products. To help participants accurately identify the product in question, the survey screen displayed an image of the product package for each set of questions and referred to a specific product name in the text. If the product package shows the manufacturer-recommended dose, this information was masked in the image provided.

To estimate DS consumption per day, they were first asked for their consumption at one time with the following question, with the appropriate unit of measurement (such as tablet or milliliter) automatically displayed depending on the product:

- We would like to ask you about the dietary supplement [product name] you have purchased in the past 3 months. Please answer the subsequent questions without looking at the product container or packaging. How much do you consume at one time? If you take it multiple times a day, please report the amount for a single intake, not the daily total.

The participants entered the quantity as an integer. Next, they were asked about their consumption frequency: “How frequently do you consume [product name]?” The frequency was selected from the following predefined 12 categories, from “more than six times per day” to “less than once per month,” as shown below. The reference period (ie, consumption in the last month) was not specified in these questions because we had already asked the participants whether they had used the DS in the previous month and whether they had used it regularly in the screening.

DS consumption per day was calculated by multiplying the number of tablets or milliliters of liquid consumed at a time by the consumption frequency. The multiplied numbers according to the consumption frequency were as follows: more than six times per day=6, five times per day=5, four times per day=4, three times per day=3, two times per day=2, once per day=1, four to six times per week=5/7, two or three times per week=2.5/7, once per week=1/7, two or three times per month=2.5/30, once per month=1/30, and less than once per month=0.5/30.

Furthermore, the duration of use, checking the manufacturer-recommended dose before use, and self-perception of their DS consumption were asked via the following questions, respectively:

- For [product’s name], how long have you been using it for? (options: <3 months, 3 months to <6 months, 6 months to <1 year, or ≥1 year)
- For [product’s name], before you started using the product, did you check the manufacturer-recommended doses of the product? (options: yes or no)
- For [product name], compared to the manufacturer-recommended doses, your consumption is… (options: more, same, or less than the manufacturer-recommended doses, unsure, or no manufacturer-recommended doses provided)

### The Manufacturer-Recommended Doses of Products

All 25 selected products indicated the manufacturer-recommended dose, although labeling of the manufacturer-recommended dose is not mandated for DS in Japan, with some exceptions [23]. Among the 25 selected products, 18 are labeled as “food with nutrient function claims (*Eiyo-Kino-Shokuhin* in Japanese),” which are required to display a manufacturer-recommended dose [24]. The other 2 products are approved as “designated quasi-drugs” by the Ministry of Health, Labour, and Welfare and are required to indicate the approved dosage and administration on the label [25]. They can be bought at a drugstore, grocery store, or convenience store without a prescription and are usually used as DS. The other 5 products were voluntarily shown the manufacturer-recommended doses. Table S1 in Multimedia Appendix 1 shows which products were labeled as “food with nutrient function claim,” “designated quasi-drugs,” or neither. The manufacturer’s website was searched using the product’s name and package image. The names and package images used in the survey were provided to researchers by INTAGE Inc. When searching the manufacturers’ websites, we ensured that the packaging images on the sites matched the packaging images of the products used in the survey. The manufacturer-recommended doses of each product were extracted from the website of the first author (MS). Although evaluating the appropriateness of the manufacturer-recommended doses was outside the scope of this study, no product exceeded the UL for any nutrient at the manufacturer-recommended doses.

### Estimation of Nutrient Intake From DSs

Nutrient intake was calculated for the following 16 nutrients using the UL established in the Dietary Reference Intakes (DRIs) for Japanese 2025 [8]: vitamins A, D, E, B_6_, niacin, folic acid, calcium, magnesium (for intakes derived from DS only), phosphorus, zinc, copper, manganese, iodine, selenium, chromium, and molybdenum. In Japanese DRIs, UL was defined based on “lowest observed adverse effect level” or “no observed adverse effect level” and uncertainty factor by the committee for DRIs composed of researchers regarding nutrition, medicine, and public health [8]. Nutrient intake from the DS was estimated by multiplying the daily DS consumption (number of tablets or mL) by the nutrient content of the products (per tablet or mL). The contents of these vitamins and minerals per tablet or milliliter in each product were obtained from the product’s website. There were no products providing phosphorus content. Thus, intakes of 15 nutrients were calculated.

### Assessment of Demographic and Lifestyle Variables

Sex and age were assessed during the screening. Other information regarding demographic and lifestyle factors was provided in the main survey. The sex at birth was selected as either male or female. Age, body weight, and body height were determined using numerical values and integers. When filling in their body weight and height, the participants were instructed to round to the nearest whole number. To prevent participants from discontinuing the survey due to their reluctance to disclose their body weight or height, these two items were not designated as mandatory responses. The following information was also collected by the questionnaire (categorization shown in parentheses): education level (junior high school, senior high school, 2-year college or technical professional school, university or higher, others, refusal to answer), employment status (unemployed, student, part-time job, full-time job), medical history (hypertension, stroke or cerebral hemorrhage, dyslipidemia, diabetes mellitus, hyperuricemia/gout, fracture or osteoporosis, liver disease, kidney disease, lung disease, heart disease, gastrointestinal disease, cancer, other, refusal to answer; multiple choice allowed); smoking status (never, past, current [<20 cigarettes per day], current [≥20 cigarettes]), drinking habit (not at all, hardly, sometimes, every day), pregnant (yes, no, refusal to answer), breastfeeding (yes, no, refusal to answer). In Japan, alcohol consumption and smoking are legally permitted from the age of 20. Therefore, following INTAGE Inc.’s policy, questions regarding drinking and smoking habits were not asked of participants aged <20 years (n=3).

Age was then categorized into 4 groups (18‐34, 35‐49, 50‐64, and ≥65 years). The ≥65-year group was classified separately to reflect the older adult population, while those aged 18‐64 years were categorized into 3 groups, approximately 15-year intervals, to capture distinct developmental and lifestyle stages. BMI (kg/m^2^) was calculated as body weight (kg) divided by the square of body height (m). Weight status was categorized as underweight (<18.5 kg/m^2^), normal (18.5‐24.9 kg/m^2^), overweight or obese (≥25 kg/m^2^) [8], or missing (who did not answer body weight, height, or both). Educational level was categorized into 4 groups: junior or senior high school, 2-year college or technical school, university or higher, and other or refusal to answer. Medical history was categorized into 3 categories: yes (having any medical history), no, and refusal to answer. Pregnancy and breastfeeding were categorized into 2 groups: yes and no/refusal. The 3 participants aged <20 years were classified as “never smokers” for smoking habits and “not at all” for alcohol consumption.

Food literacy associated with adherence to the manufacturer-recommended regimen was assessed using the Japanese version of the Self-Perceived Food Literacy Scale [26]. The Japanese version was developed and used in a previous study [27]. This scale is an expert-based, theory-driven, and validated tool for measuring food literacy in healthy eating that focuses on 8 domains. Participants were asked to respond on a 5-point Likert scale (1=not at all or never; 5=yes or always). The food literacy score was calculated as the average of all 29 items. Negative items were reversed in the calculation. Therefore, the higher the score, the higher the food literacy. Possible scores ranged from 1 to 5. Participants were categorized into 2 groups based on their scores: those below the median and those at or above the median.

### Analyzed Participants

Participants were excluded from the analysis if their amount of DS consumption was extremely high due to the possibility of incorrect responses, specifically those who reported taking a 60- or 90-day package more than 6 times or more per day (ie, ≥360 or ≥540 tablets per day) (n=2). Daily consumption of DS compared to the recommended dose was calculated as the dietary consumption of DS (ie, the number of tablets or mL of liquid per day) divided by the manufacturer-recommended daily dose of the product. When the daily consumption of DS compared to the recommended dose (%) was over 100, the participants were categorized into the “exceeding–manufacturer-recommended-dose” group (who consumed DS exceeding the recommended dose). Others were classified into the “within–manufacturer-recommended-dose” group (who consumed DS at the same or below the recommended dose).

### Statistical Analysis

All statistical analyses were performed using the SAS statistical software (version 9.4; SAS Institute Inc). Significant results were defined as 2-tailed *P* values <.05. Variables of demographic and lifestyle factors and food literacy scores are presented as frequencies and percentages for categorical variables and means and SDs for continuous variables. First, these variables were compared between 2 groups: the within–manufacturer-recommended-dose group and exceeding–manufacturer-recommended-dose group. Statistical differences were analyzed using the Student *t* test for continuous variables and the chi-square test for categorical variables.

Univariate and multivariate logistic regression models were used to evaluate the factors associated with DS consumption exceeding manufacturer-recommended doses. Individuals in the manufacturer-recommended-dose group served as the reference group. The explanatory variables were demographic and lifestyle variables, namely, age, sex, weight status, educational level, employment status, medical history, smoking status, and drinking habits. In addition, the following variables were examined as explanatory variables: form of DS, featured nutrients of DS tablets, duration of use, checking of the manufacturer-recommended dose before use, self-perception of DS consumption, and food literacy score. Featured nutrients of tablet form DS were categorized as follows: “multivitamin/multimineral” containing 3 or more vitamins or minerals; “folic acid and iron” containing folic acid and iron as the primary nutrients; “single fat-soluble vitamins” containing <3 fat-soluble vitamins as the primary nutrients; “single water-soluble vitamins” containing <3 water-soluble vitamins as the primary nutrients; and “single mineral” containing <3 minerals as the primary nutrients.

In the multivariate model, the following covariates were mutually adjusted for: sex, age (18‐34, 34‐49, 50‐64, or ≥65 years), weight status (<18.5 kg/m^2^, 18.5‐24.9 kg/m^2^, ≥25 kg/m^2^, or missing), educational level (junior or senior high school, 2-year college or technical professional school, university or higher, or others or refusal to answer), employment status (unemployed, student, part-time job, or full-time job), medical history (yes, no, or refusal to answer), smoking status (never, past, current [<20 cigarettes a day], or current [≥20 cigarettes a day]), drinking habit (not at all, hardly, sometimes, or every day), and form of DS (liquid or tablet). The form of the DS was adjusted for differences in user characteristics, including consumption compared to the manufacturer-recommended doses, sex, weight status, and whether users checked the manufacturer-recommended doses before use (Table S2 in Multimedia Appendix 1). When one of the main variables, such as the duration of DS use, checking the manufacturer-recommended dose before use, and self-perception of DS consumption compared with the manufacturer-recommended dose, or food literacy score, was included in the model, it was analyzed alongside 9 covariates. When the featured nutrients of tablet form DS were the main variables, the form of DS was not adjusted because this analysis only included participants who consumed tablet-type DS. Additionally, linear trends of odds ratios were tested for age, educational level, employment status, smoking status, drinking habit, and duration of DS use. In the regression analysis, ordinal variables (ie, 1, 2, 3, and 4) were used as explanatory variables for educational level, employment status, smoking status, and drinking habit. The median value of each category was used for age and duration of use.

Nutrient intake from DS was described as the median and 25th and 75th percentiles for those with ULs. The medians and 25th and 75th percentiles were calculated for those who consumed DS containing each nutrient with ULs. For example, vitamin A intake was described in participants who consume DS containing vitamin A. In other words, participants who consumed DS without vitamin A were excluded from the calculation of vitamin A intake. In addition, the number and proportion of participants who exceeded the UL were calculated for all participants and within– or exceeding–manufacturer-recommended-dose group.

For sensitivity analysis, the same analysis was conducted after excluding participants who reported consuming DS five times or more than the manufacturer-recommended dose. Overall, 1821 participants were analyzed after excluding those who consumed DS five times or more than the manufacturer-recommended dose (n=183).

## Results

**Figure 2.**
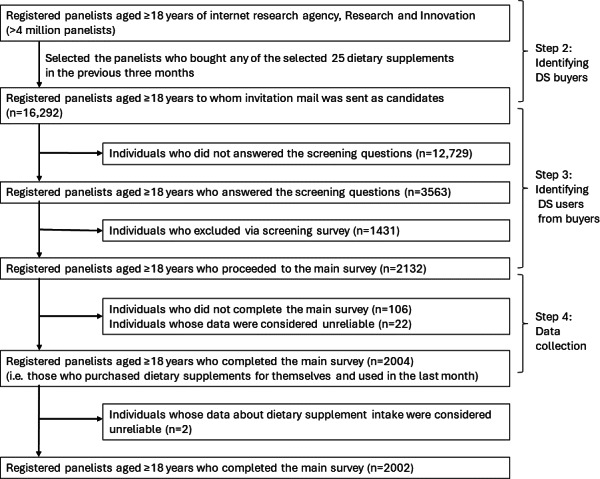
Flowchart of study participants’ selection. In step 2, those with a record of purchasing 2 or more selected DS products were randomly assigned as potential respondents to only one of the selected DS products. DS: dietary supplement.

A flow diagram of the participant selection process is shown in Figure 2. The mean age was 43.7 (SD 10.7) years, and half of the participants were aged 34‐49 years (Table 1). The mean BMI was 21.6 (SD 3.6) kg/m^2^ (n=1755, for those who answered both body weight and height); 59.5% (1192/2002) of the participants were categorized as having normal weight, while 12.3% (247/2002) did not answer their body weight, height, or both. The participants were predominantly female (1514/2002, 75.6%). The mean age and BMI were not significantly different between the within– and exceeding–manufacturer-recommended-dose groups (43.6 [SD 10.6] vs 44.2 [SD 11.1] years; 21.6 [SD 3.6] vs 21.8 [SD 3.4] kg/m^2^, respectively). The exceeding–manufacturer-recommended-dose group had a higher proportion of participants aged 51‐64 years and with drinking habits “every day.” There were no statistically significant differences in other demographic and lifestyle variables between the within– and exceeding–manufacturer-recommended-dose groups.

**Table 1.** Characteristics of dietary supplement users (n=2002) according to dietary supplement consumption compared to the recommended dose^a^.

Variables	All (n=2002), n (%)	Within–manufacturer-recommended dose^b^ (n=1631), n (%)	Exceeding–manufacturer-recommended dose^c^ (n=371), n (%)	*P* value^d^
Age (years)				.04
18‐34	421 (21.0)	337 (20.7)	84 (22.6)	
34‐49	956 (47.8)	803 (49.2)	153 (41.2)	
50‐64	567 (28.3)	444 (27.2)	123 (33.2)	
≥65	58 (2.9)	47 (2.9)	11 (3.0)	
Weight status				.35
Underweight (BMI<18.5 kg/m^2^)	298 (14.9)	253 (15.5)	45 (12.1)	
Normal (BMI 18.5‐24.9 kg/m^2^)	1192 (59.5)	961 (58.9)	231 (62.3)	
Overweight or obesity (BMI≥25 kg/m^2^)	265 (13.2)	213 (13.1)	52 (14.0)	
Missing	247 (12.3)	204 (12.5)	43 (11.6)	
Sex				.03
Male	488 (24.4)	381 (23.4)	107 (28.8)	
Female	1514 (75.6)	1250 (76.6)	264 (71.2)	
Educational level				.27
Junior or senior high school	670 (33.5)	539 (33.0)	131 (35.3)	
2-year college or technical school	588 (29.4)	472 (28.9)	116 (31.3)	
University or higher	669 (33.4)	561 (34.4)	108 (29.1)	
Others or refusal to answer	75 (3.7)	59 (3.6)	16 (4.3)	
Employment status				.05
Unemployed	496 (24.8)	424 (26.0)	72 (19.4)	
Student	27 (1.3)	20 (1.2)	7 (1.9)	
Part-time job	533 (26.6)	427 (26.2)	106 (28.6)	
Full-time job	946 (47.3)	760 (46.6)	186 (50.1)	
Medical history				.59
No	1276 (63.7)	1034 (63.4)	242 (65.2)	
Yes	629 (31.4)	520 (31.9)	109 (29.4)	
Refusal to answer	97 (4.8)	77 (4.7)	20 (5.4)	
Smoking status				.05
Never	1358 (67.8)	1125 (69.0)	233 (62.8)	
Past	369 (18.4)	297 (18.2)	72 (19.4)	
Current (<20 cigarettes a day)	219 (10.9)	168 (10.3)	51 (13.7)	
Current (≥20 cigarettes a day)	56 (2.8)	41 (2.5)	15 (4.0)	
Drinking habit				.002
Not at all	647 (32.3)	524 (32.1)	123 (33.2)	
Hardly	478 (23.9)	411 (25.2)	67 (18.1)	
Sometimes	616 (30.8)	501 (30.7)	115 (31.0)	
Every day	261 (13.0)	195 (12.0)	66 (17.8)	
Pregnant (n, % of females)				.89
Yes	42 (2.8)	35 (2.8)	7 (2.7)	
No or refusal to answer	1467 (97.2)	1215 (97.2)	257 (97.3)	
Breastfeeding (n, % of females)				.16
Yes	48 (3.2)	36 (2.9)	12 (4.5)	
No or refusal to answer	1461 (96.8)	1214 (97.1)	252 (95.5)	
Form of dietary supplement				<.001
Liquid	301 (15.0)	287 (17.5)	14 (3.8)	
Tablet	1702 (85.0)	1345 (82.5)	357 (96.2)	
Featured nutrients of the dietary supplement^e^ (tablet form only)				<.001
Multivitamins/multimineral	486 (24.3)	394 (24.2)	92 (24.8)	
Folic acid and iron	129 (6.4)	108 (6.6)	21 (5.7)	
Single fat-soluble vitamins	169 (8.4)	132 (8.1)	37 (10.0)	
Single water-soluble vitamins	470 (23.5)	347 (21.3)	123 (33.2)	
Single minerals	447 (22.3)	363 (22.3)	84 (22.6)	
Duration of the dietary supplement use				<.001
<3 months	484 (24.2)	420 (25.8)	64 (17.3)	
3 months to <6 months	289 (14.4)	245 (15.0)	44 (11.9)	
6 months to <1 year	375 (18.7)	303 (18.6)	72 (19.4)	
≥1 year	855 (42.7)	664 (40.7)	191 (51.5)	
Checking the manufacturer-recommended dose before use: “Before you started using the product, did you check the recommended dose of the product?”				<.001
Yes	1686 (84.2)	1351 (82.8)	335 (90.3)	
No	316 (15.8)	280 (17.2)	36 (9.7)	
Self-perception of their dietary supplement consumption: “Compared to the recommended dose, your consumption is...”				<.001
More than the recommended dose	82 (4.1)	25 (1.5)	57 (15.4)	
Same as the recommended dose	1268 (63.3)	1056 (64.7)	212 (57.1)	
Less than the recommended dose	240 (12.0)	200 (12.3)	40 (10.8)	
Unsure	387 (19.3)	326 (20.0)	61 (16.4)	
No recommended dose provided	25 (1.2)	24 (1.5)	1 (0.3)	
Food literacy score^f^				.49
Less than median score	1020 (50.9)	837 (51.3)	183 (49.3)	
Same as the median score or more	982 (49.1)	794 (48.7)	188 (50.7)	

a Participants were panelists who bought one of the 25 selected dietary supplements in the previous 3 months and used it within the past month or regularly. Information was collected from one of the 25 dietary supplements used by each participant.

b Those whose daily intake of the dietary supplement was equal to or below the manufacturer-recommended dose.

c Those whose intake exceeded the manufacturer-recommended dose.

d *P* values by chi-square test for categorical variables. Statistical significance was set at *P*<.05.

e “Multivitamins” contained 3 or more vitamins or minerals. “Folic acid and iron” contained folic acid and iron as the primary nutrients. “Single fat-soluble vitamins” contained <3 fat-soluble vitamins as the primary nutrients. “Single water-soluble vitamins” contained <3 water-soluble vitamins as the primary nutrients. “Single mineral” contained <3 minerals as the primary nutrients.

f The median score among the participants was 3.17, with possible scores ranging from 1 to 5.

Among 2002 participants, 18.5% (371/2002) exceeded the manufacturer-recommended dose. Regarding the variables related to DS use, the exceeding–manufacturer-recommended-dose group had a higher proportion of individuals who used tablet-form DS, especially single water-soluble vitamin products, and those who used the products for ≥6 months. In addition, the proportion of participants who checked the manufacturer-recommended doses was higher in the exceeding–manufacturer-recommended-dose DS group than in the within–manufacturer-recommended-dose group (90.3% [335/371] vs 82.8% [1351/1631]). The proportion of participants who thought their consumption was “more than the manufacturer-recommended dose” was higher in the exceeding–manufacturer-recommended group (57/371, 15.4%) than in the within–manufacturer-recommended group (25/1631, 1.5%). Moreover, 57.1% (212/371) and 10.8% (40/371) of those in the exceeding–manufacturer-recommended-dose group thought that their consumption was the same or below the recommended dose, respectively. In the exceeding–manufacturer-recommended-dose group, the daily DS consumption compared with the manufacturer-recommended dose (%) ranged from 394% for single water-soluble vitamin tablets to 543% for folic acid and iron tablet DS (Table S3 in Multimedia Appendix 1).

The unadjusted and adjusted odds ratios (AORs) for exceeding the manufacturer-recommended dose are presented in Table 2. From the multivariate logistic regression analysis, participants aged 50‐64 years (AOR 1.45, 95% CI 1.10-1.92), those with part-time jobs (AOR 1.58, 95% CI 1.13-2.21), or those with full-time jobs (AOR 1.49, 95% CI 1.09-2.03) were likely to consume DS exceeding the manufacturer-recommended doses compared with those aged 18‐34 years and those who were unemployed. In addition, participants who used a tablet form DS (AOR 6.02, 95% CI 3.46-10.47), especially a single water-soluble vitamin tablet DS (AOR 1.51, 95% CI 1.11-2.06), were likely to exceed the manufacturer-recommended doses compared to those using liquid-form DS and multivitamin/multimineral tablets. Moreover, using DS for 6 months to <1 year or ≥1 year, and those who thought they took “more than” the manufacturer-recommended doses were associated with exceeding the manufacturer-recommended doses.

**Table 2.** Factors associated with dietary supplement consumption exceeding the manufacturer-recommended doses among 2002 dietary supplement users^a^.

Variables	Crude model^b^		Adjusted model^c^	
	OR^d^ (95% CI)	*P* for trend	AOR^e^ (95% CI)	*P* for trend
Age (years)		.26		.23
18‐34	1.26 (0.93‐1.70)		1.20 (0.88‐1.63)	
34‐49	1.00 (Reference)		1.00 (Reference)	
50‐64	1.40 (1.07‐1.83)		1.43 (1.08‐1.90)	
≥65	1.25 (0.64‐2.47)		1.35 (0.67‐2.72)	
Weight status^f^		—^g^		
Underweight (BMI<18.5 kg/m^2^)	0.73 (0.51‐1.04)		0.72 (0.50‐1.04)	
Normal (BMI 18.5‐24.9 kg/m^2^)	1.00 (Reference)		1.00 (Reference)	
Overweight or obesity (BMI≥25 kg/m^2^)	0.97 (0.69‐1.37)		0.95 (0.66‐1.37)	
Missing	0.89 (0.62‐1.28)		0.94 (0.64‐1.36)	
Sex		—^g^		—^g^
Male	1.00 (Reference)		1.00 (Reference)	
Female	0.83 (0.64‐1.07)		0.84 (0.63‐1.11)	
Educational level		.24		.26
Junior or senior high school	1.00 (Reference)		1.00 (Reference)	
2-year college or technical school	1.05 (0.79‐1.39)		1.12 (0.83‐1.50)	
University or higher	0.79 (0.59‐1.05)		0.78 (0.58‐1.05)	
Others or refusal to answer	1.18 (0.66‐2.12)		1.25 (0.68‐2.29)	
Employment status		.02		.01
Unemployed	1.00 (Reference)		1.00 (Reference)	
Student	2.12 (0.86‐5.20)		2.12 (0.83‐5.40)	
Part-time job	1.46 (1.05‐2.04)		1.56 (1.12‐2.19)	
Full-time job	1.40 (1.04‐1.90)		1.47 (1.07‐2.02)	
Medical history		—^g^		—^g^
Yes	0.87 (0.67‐1.12)		0.83 (0.64‐1.09)	
No	1.00 (Reference)		1.00 (Reference)	
Refusal to answer	0.97 (0.56‐1.67)		1.00 (0.57‐1.74)	
Smoking status		.006		.12
Never	1.00 (Reference)		1.00 (Reference)	
Past	1.17 (0.87‐1.58)		0.99 (0.72‐1.36)	
Current (<20 cigarettes a day)	1.39 (0.98‐1.98)		1.30 (0.90‐1.90)	
Current (≥20 cigarettes a day)	1.58 (0.83‐3.00)		1.28 (0.65‐2.50)	
Drinking habit		.07		.27
Not at all	1.00 (Reference)		1.00 (Reference)	
Hardly	0.68 (0.49‐0.95)		0.67 (0.48‐0.94)	
Sometimes	0.97 (0.73‐1.29)		0.98 (0.73‐1.32)	
Every day	1.37 (0.96‐1.94)		1.20 (0.83‐1.75)	
Form of dietary supplement		—^g^		—^g^
Liquid type	1.00 (Reference)		1.00 (Reference)	
Tablet type	6.12 (3.39‐11.03)		6.67 (3.69‐12.07)	
Featured nutrients of the dietary supplement^h^ (tablet type only)		—^g^		—^g^
Multivitamins/multimineral	1.00 (Reference)		1.00 (Reference)	
Folic acid and iron	0.87 (0.52‐1.47)		0.99 (0.58‐1.69)	
Single fat-soluble vitamins	1.19 (0.77‐1.84)		1.18 (0.76‐1.84)	
Single water-soluble vitamins	1.54 (1.13‐2.10)		1.52 (1.11‐2.09)	
Single minerals	1.00 (0.72‐1.40)		0.95 (0.68‐1.33)	
Duration of the dietary supplement use		<.001		<.001
<3 months	1.00 (Reference)		1.00 (Reference)	
3 months to <6 months	1.16 (0.76‐1.77)		1.13 (0.74‐1.72)	
6 months to <1 year	1.52 (1.04‐2.22)		1.54 (1.06‐2.23)	
≥1 year	1.94 (1.42‐2.65)		1.67 (1.22‐2.30)	
Checking the manufacturer-recommended dose before use: “Before you started using the product, did you check the recommended dose of the product?”		—^g^		—^g^
Yes	0.53 (0.36‐0.76)		1.20 (0.78‐1.85)	
No	1.00 (Reference)		1.00 (Reference)	
Self-perception of their dietary supplement consumption: “Compared to the recommended dose, your consumption is...”		—^g^		—^g^
More than the recommended dose	10.77 (6.55‐17.73)		11.46 (6.83‐19.21)	
Same as the recommended dose	1.00 (Reference)		1.00 (Reference)	
Less than the recommended dose	0.89 (0.60‐1.31)		0.89 (0.60‐1.31)	
Unsure	0.92 (0.67‐1.26)		1.36 (0.97‐1.90)	
No recommended dose provided	0.21 (0.03‐1.57)		0.37 (0.05‐2.83)	
Food literacy score^i^		—^g^		—^g^
Less than median score	1.00 (Reference)		1.00 (Reference)	
Same as median score or more	1.09 (0.87‐1.37)		1.07 (1.07‐0.84)	

a Participants were panelists who bought one of the 25 selected dietary supplements in the previous 3 months and used it within the past month or regularly. Information was collected from one of the 25 dietary supplements used by each participant.

b Derived from univariate logistic regression models.

c Derived from multivariable logistic regression models mutually adjusted for sex, age, weight status, educational level, employment status, medical history, smoking status, drinking habit, and form of dietary supplement; excluded for main variables.

d OR: odds ratio.

e AOR: adjusted odds ratio.

f “Multivitamins/multimineral” contained 3 or more vitamins or minerals. “Folic acid and iron” contained folic acid and iron as the primary nutrients. “Single fat-soluble vitamins” contained <3 fat-soluble vitamins as the primary nutrients. “Single water-soluble vitamins” contained <3 water-soluble vitamins as the primary nutrients. “Single mineral” contained <3 minerals as the primary nutrients.

g The *P* for trend was not calculated, since a linear relationship between categories could not be assumedor because only one exposure category (in addition to the reference group) was included..

h Users of the liquid-form dietary supplement were excluded from the analysis. The form of the dietary supplement was not adjusted.

i The median score among participants was 3.17, with possible scores ranging from 1 to 5.

Table 3 describes the intake of 15 nutrients with UL as defined in the DRIs for Japanese, along with the number of participants taking DSs containing these nutrients. Among 2002 participants, 1705 adults took DS containing ≥1 nutrient with UL. Among 371 adults in the exceeding–manufacturer-recommended-dose group, 297 adults took DS containing ≥1 nutrient with UL. No participant in the within–manufacturer-recommended-dose group exceeded the UL for their DS intake. In the exceeding–manufacturer-recommended-dose group, 61.9% (184/297) exceeded the UL for at least one nutrient. For vitamin A, niacin, folic acid, and zinc, the proportion of users who exceed UL was more than 40% in the exceeding–manufacturer-recommended-dose group using DS containing the nutrient with UL, namely, 50.7% (37/73) for vitamin A, 57% (73/128) for niacin, 48.1% (76/158) for folic acid, 59% (26/44) for magnesium, 52.9% (46/87) for zinc.

**Table 3.** Nutrient intake with defined UL^a^ among dietary supplement users (n=2002)^b^.

Nutrients	UL^c^	All (n=2002)		Within–manufacturer-recommended dose^d^ (n=1631)			Exceeding–manufacturer-recommended dose^e^ (n=371)		
		Participants^f^, n	Exceed UL, n (%)	Participant^f^s, n	Nutrient intake, median (IQR)	Exceed UL, n (%)	Participants^f^, n	Nutrient intake, median (IQR)	Exceed UL, n (%)
Vitamin A (μg)	2700	400	37 (9.3)	327	770 (450-770)	0 (0)	73	3080 (1800‐5400)	37 (50.7)
Vitamin D (μg)	100	648	15 (2.3)	527	6.6 (5.0‐10.0)	0 (0)	121	33 (20.0‐60.0)	15 (12.4)
Vitamin E (mg)	Men: 800 Women aged 18-29 years: 650 Women aged 30-74 years: 700	466	3 (0.6)	381	6.3 (6.3‐10.0)	0 (0)	85	37.8 (18.9‐54.0)	3 (3.5)
Niacin^g^ (mg)	Men aged 18‐29 years and 65-74 years: 80 Men aged 30‐64 years: 85 Women: 65	874	73 (8.4)	746	13 (2.1‐15.0)	0 (0)	128	80 (57.0‐104.0)	73 (57.0)
Vitamin B_6_ (mg)	Men aged 18‐29 years and 65-74 years: 55 Men aged 30‐64 years: 60 Women: 45	943	39 (4.1)	805	1.3 (0.7‐3.2)	0 (0)	138	16 (7.8‐60.0)	39 (28.3)
Folic acid (μg)	Aged 18‐29 years, ≥65 years: 900 Aged 30‐64 years: 1000	825	76 (9.2)	667	200 (100-240)	0 (0)	158	980 (400‐1440)	76 (48.1)
Calcium (mg)	2500	270	9 (3.3)	216	200 (90-360)	0 (0)	54	735 (450‐1500)	9 (16.7)
Magnesium (mg)	350^h^	201	26 (12.9)	157	125 (100-250)	0 (0)	44	493 (250-824)	26 (59.0)
Zinc (mg)	Men aged 18‐29 years: 40 Men aged 30‐74 years: 45 Women: 35	435	46 (10.6)	348	15 (6.0‐15.0)	0 (0)	87	45 (18.0‐90.0)	46 (52.9)
Copper (mg)	7	125	2 (1.6)	94	0.6 (0.2‐0.6)	0 (0)	31	1.2 (0.8‐3.6)	2 (6.5)
Manganese (mg)	11	90	1 (1.1)	68	1.5 (0.9‐1.5)	0 (0)	22	3.3 (2.2‐6.5)	1 (4.5)
Iodine (μg)	3000	90	0 (0.0)	68	45.0 (25.6‐50.8)	0 (0)	22	102 (68-162)	0 (0.0)
Selenium (μg)	Men aged 18‐29 years: 400 Men aged 30‐74 years: 450 Women: 350	404	5 (1.2)	321	50 (30.2‐50.0)	0 (0)	83	250 (100-300)	5 (6.0)
Chromium (μg)	500	404	4 (1.0)	321	60 (21.4‐60.0)	0 (0)	83	180 (85-360)	4 (4.8)
Molybdenum (μg)	Men: 600 Women: 500	90	0 (0.0)	68	9.0 (5.2‐10.5)	0 (0)	22	21 (14.0‐32.4)	0 (0.0)
≥1 nutrients above		1705	184 (10.8)	1416	—	0 (0)	297	—	184 (61.9)

a UL: tolerable upper intake level.

b Participants were panelists who had bought one of the 25 selected dietary supplements in the last 3 months and used it within the past month or regularly. Information was collected from one of the 25 dietary supplements used by each participant.

c UL is defined by the Dietary Reference Intakes for Japanese, 2025.

d Those whose daily intake of the dietary supplement was equal to or below the manufacturer-recommended amount.

e Those whose intake exceeded the manufacturer-recommended amount.

f Number of participants taking nutrient-containing dietary supplements in the leftmost column.

g Amount of nicotinamide.

h ULs were defined for intakes derived from dietary supplements only.

For sensitivity analysis, 10.4% (190/1821) of patients were categorized into the exceeding–manufacturer-recommended-dose group. The results for the factors associated with DS consumption exceeding the manufacturer-recommended doses were almost unchanged from the original analysis of the 2002 participants (Tables S4 and S5 in Multimedia Appendix 1). The number of participants who consumed DS containing ≥1 nutrients with ULs was 134 adults. Of these, the number of participants who exceeded UL for at least one nutrient with ULs was 44 (32.8%). These numbers were much smaller than those included in the main analysis (Table S6 in Multimedia Appendix 1).

## Discussion

### Principal Findings

To the best of our knowledge, this is the first study to examine the factors associated with DS consumption exceeding the manufacturer-recommended doses among Japanese adults. Among 2002 DS users, 371 (18.5%) consumed DS that exceeded the manufacturer-recommended doses indicated on the package. Consumption of DS above the recommended dose was associated with middle age, part-time or full-time employment, use of tablet-form DS, especially single water-soluble vitamin tablets, use of DS for 6 months or more, and conscious consumption above the recommended dose. Among 1705 individuals consuming DS containing nutrients with ULs, 17.4% (n=297) exceeded the recommended dose. Of these, 61.9% (184/297) of individuals surpassed the UL by at least one nutrient. The results will contribute to a more accurate understanding of DS overuse behaviors, their associated factors, and possible health outcomes.

In this study, the proportion of participants who exceeded the manufacturer-recommended dose was 371 (18.5%) out of 2002, which is substantially higher than that previously reported (2.4%) [20]. When the proportion was calculated based on the self-perception of DS consumption, 4.1% ([25+57]/2002) exceeded the manufacturer-recommended dose, which is close to that previously reported. Among the 371 participants, only 15.4% (57/371) were aware that their consumption exceeded the recommended dose. These findings suggest that self-perception of adherence to the recommended dose is not a reliable indicator of actual dose consumption. To accurately assess compliance with the recommended doses, identifying the specific product used and comparing the reported consumption with the corresponding manufacturer-recommended dose are essential.

Previous research on DS overuse has focused on the use of multiple DS products [28,29], and to our knowledge, no studies have specifically examined DS consumption exceeding the manufacturer-recommended dose. Therefore, although the reasons for the observed associations with the abovementioned factors remain speculative, several possible explanations can be considered. Among older adults, a higher prevalence of multiple DS use has been reported in the US population [28,29]. As health deterioration associated with aging may increase interest in diet [30], older individuals may consciously or unconsciously consume higher amounts of DS. Regarding employment status, individuals with part-time or full-time jobs may rely on DS as a convenient means of supplementing their nutrition due to busy schedules, which could lead to excessive DS intake. A previous study has reported that individuals working long hours often perceive barriers to consuming healthy meals [31]. Since only 31.4% (627/2002) of participants reported a medical history in this study, and 70% of DS users among patients did not disclose DS use to their physicians in a previous study [32], it was unlikely that participants had exceeded the manufacturer-recommended dose of DS based on specialists’ recommendations.

Users of the DS tablet form, especially those using a single water-soluble vitamin tablet, were more likely to exceed the manufacturer-recommended dose. In addition, use of DS for 6 months or more, and conscious consumption above the recommended dose were also associated. With respect to the form of DS, liquid DS may be used more episodically and may be less likely to result in use above the recommended dose, as a previous study suggested that energy drinks are used episodically rather than regularly among college students [33]. According to a US study, DS of water-soluble vitamins were used to expect that it would boost the immune system, prevent colds, and improve overall health [34]. Some participants may have intentionally consumed more than the manufacturer-recommended dose regularly, believing that it would yield greater health benefits, even when they were aware of the manufacturer-recommended dosages. Moreover, a considerable proportion of individuals may have either misinterpreted or failed to retain the correct information regarding the recommended intake even after reviewing it. For such individuals, even if dosage guidelines are clearly indicated on labels, the information may not be effectively used. Notably, no significant differences in food literacy scores were found between those who adhered to appropriate dosage levels and those who consumed higher dosage levels. This finding implies that general food literacy scales may not adequately capture the literacy specific to DS use. Alternatively, factors beyond literacy, such as health beliefs, perceived efficacy, and trust in DS, may contribute to excessive intake. To prevent overconsumption of DSs, it may be necessary to improve the clarity and accessibility of dosage-related information and increase public awareness of the potential harm associated with excessive intake.

In this study, among 1705 participants who consumed DS containing nutrients with UL, 10.7% (184/1705) of participants exceeded the UL for at least one nutrient. They were asked about their consumption of only one DS product. Although some participants may have obtained nutrients from other DS sources, this was not captured in the survey. Previous studies in the United States reported that the concurrent use of multiple DS was common [28,29]. For example, middle-aged and older DS users use an average of more than 3.2 different DS, with 41.9% using 4 or more products [28]. Another US study on adults found that all individuals whose usual daily folic acid intake exceeded the UL consumed folic acid from multiple sources [16]. Consequently, among our study participants, the proportion of individuals exceeding the UL for specific nutrients may have been higher if nutrient intake from multiple concurrently used DS had been considered.

Previous studies have reported the proportion of individuals exceeding the UL. In the United States, approximately 20%‐40% of adults use vitamin DSs, with reported rates of exceeding UL at 7% for folic acid, 3% for vitamin A, 3.5% for vitamin B_6_, and 1.6% for vitamin C [13]. Approximately 7%‐12% of DS users exceeded the UL for iron or zinc, and less than 3%‐7% exceeded the UL for calcium or magnesium [11]. In the Netherlands, although the nutrient intake from both DSs and fortified foods exceeded the UL for some participants, the proportions were <3% for calcium, iron, vitamins B_6_, vitamin A, and folate [14]. In Canada, when intake from DS was included, ≥10% of individuals in certain age and sex groups had intakes of vitamins A and C, niacin, folic acid, iron, zinc, and magnesium that exceeded the UL [15]. However, few studies have provided detailed descriptions of how individuals use DS, such as whether they use multiple DS concurrently or whether they consume doses exceeding the manufacturer-recommended dose. Therefore, future studies should not only report the proportion of individuals exceeding the UL but also examine the behavioral patterns of DS use to better understand the factors contributing to excessive nutrient intake.

### Strengths and Limitations

The strength of this study lies in the use of purchase history data to recruit participants. We targeted panelists who purchased and used specified DS products. This approach helped minimize misreporting related to product identification, such as when respondents were unable to recall or report product names accurately. In addition, presenting the specific unit for the product in the questionnaire might reduce the misreporting of the dosage, although some misreporting appeared. Consequently, this enabled a reliable comparison between the participants’ reported DS consumption and the recommended dosage for each product. However, this study has some limitations. First, the participants were limited to panelists registered as online survey monitors, which may compromise the generalizability of the findings owing to potential sampling bias. Our participants had higher education levels than the nationally representative sample aged 20‐74 years (43.2% for junior or senior high school, 15.6% for 2-year college or technical school, 24.2% for university or higher; education level reported in the Population Census of Japan 2020 [35]). Second, the extent to which the DS included in this study represents the full range of products consumed by Japanese DS users remains unclear and may be due to the lack of comprehensive and nationwide statistical data on the consumption of DS products. However, as the items were selected based on their high purchase frequency in transaction records, they were considered to ensure a certain level of representativeness. Third, data on daily DS consumption and frequency of consumption were self-reported, which introduces the possibility of reporting errors. Although the survey was designed to facilitate accurate responses by clearly specifying the units of each product on the response screen, some participants likely misreported their consumption quantities. We excluded those who consumed extremely high doses of DS (≥360 tablets per day) from the analysis because they were likely to have overreported it; however, some participants in the analyzed group may have also overreported it. However, when we excluded participants who consumed 5 times the manufacturer-recommended doses, the results were similar (Table S1 in Multimedia Appendix 1). Fourth, this study did not assess participants’ concurrent use of multiple DS. To ensure the feasibility of the survey and obtain accurate responses regarding the selected products, participants with purchase histories of multiple DS products were randomly assigned to answer questions about only one of those products. Consequently, information on the use of multiple DS and their contributions to overall nutrient intake and the adequacy of nutrient intake remains unknown. If the concurrent use of multiple DS is also considered, the proportion of participants exceeding the UL may increase further, indicating the need for greater public awareness and caution regarding DS use. Further studies are required to better understand the patterns of multiple DS use and their implications for total nutrient consumption.

### Conclusions

In conclusion, middle-aged, part-time or full-time jobs, using tablet-form DS, and using DS for 6 months or more were associated with DS consumption exceeding the manufacturer-recommended dose, which may lead to excessive nutrient intake. Some individuals consume excessive doses of DSs, highlighting the need to raise awareness about the potential health risks associated with overconsumption.

## Supplementary material

10.2196/82623 Multimedia Appendix 1 Additional tables describing analyses of products and participants, along with sensitivity analyses.

## Abbreviations

AOR: adjusted odds ratio
CHERRIES: Checklist for Reporting Results of Internet e-Surveys
DRI: Dietary Reference Intakes for Japanese
DS: dietary supplement
UL: tolerable upper intake level
