# Supplementary material for: Prevalence and Associated Factors of Excessive Dietary Supplement Use Among Japanese Adults: Cross-Sectional Study
Source: Interact J Med Res. 2026 Mar 19;15:e82623. doi: 10.2196/82623 (PMC13002161; doi:10.2196/82623)
Supplement: Multimedia Appendix 1 [file ijmr-v15-e82623-s001.docx]

# **Table S1.** Selected dietary supplements and target sample size.

| Categories for selection | Product name (English) | Product name (Japanese) | Target sample size (N) | Respondents (N) | Analysed participants (N)* | Category for analysis |
| --- | --- | --- | --- | --- | --- | --- |
| Liquid |  |  | **300** |  |  |  |
|  | Taiho Pharmaceutical, Tiovita Drink 100ml† | 大鵬薬品, チオビタ・ドリンク 100ml | 150 | 152 | 152 | Liquid |
|  | Alinamin, Alinamin Medical Balance 100ml† | アリナミン,アリナミンメディカルバランス 100ml | 150 | 149 | 149 | Liquid |
| Tablet, multivitamins |  |  | **400** |  |  |  |
|  | DHC, Multivitamin (60-day supply) ‡ | DHC,マルチビタミン（60日分） | 120 | 120 | 120 | Tablet-multivitamins/minerals |
|  | Asahi, Dear-Natura Style, Iron and Multivitamin (60-day supply) ‡ | アサヒ,ディアナチュラスタイル, 鉄マルチビタミン（60日分） | 115 | 116 | 115 | Tablet-multivitamins/minerals |
|  | Asahi, Dear-Natura Style, Multivitamin (60-day supply) ‡ | アサヒ, ディアナチュラスタイル, マルチビタミン（60日分） | 60 | 60 | 60 | Tablet-multivitamins/minerals |
|  | Otsuka Pharmaceutical, Nature Made, Super Multivitamin & Mineral‡ | 大塚製薬, ネイチャーメイド, スーパーマルチビタミン＆ミネラル | 35 | 35 | 35 | Tablet-multivitamins/minerals |
|  | DHC, Multivitamin/mineral + Q10‡ | DHC, マルチビタミン／ミネラル＋Q10 | 35 | 35 | 35 | Tablet-multivitamins/minerals |
|  | Asahi, Dear-Natura Style, Iron and Multivitamin (20-day supply) ‡ | アサヒ, ディアナチュラスタイル, 鉄マルチビタミン（20日分） | 35 | 35 | 35 | Tablet-multivitamins/minerals |
| Tablet, fat-soluble vitamins |  |  | **200** |  |  |  |
|  | DHC, Vitamin D (60-day supply) | DHC, ビタミンD（60日分） | 55 | 55 | 55 | Tablet-single fat-soluble vitamins |
|  | DHC, 60 Day Natural Vitamin E [Soy] (60-day supply) | DHC, 60日天然ビタミンE[大豆] （60日分） | 40 | 41 | 41 | Tablet-single fat-soluble vitamins |
|  | Otsuka Pharmaceutical Nature Made Super Vitamin D | 大塚製薬, ネイチャーメイド, スーパービタミンD | 25 | 23 | 23 | Tablet-single fat-soluble vitamins |
|  | Asahi, Dear-Natura, Vitamin D‡ | アサヒ, ディアナチュラ, ビタミンD | 25 | 25 | 25 | Tablet-single fat-soluble vitamins |
|  | Asahi, Dear-Natura, Vitamin E (60-day supply) ‡ | アサヒ, ディアナチュラ, ビタミンE（60日分） | 15 | 15 | 15 | Tablet-single fat-soluble vitamins |
|  | Daiso Industries, Turmeric + Vitamin E | 大創産業, ウコン＋ビタミンE | 10 | 10 | 10 | Tablet-single fat-soluble vitamins |
|  | Asahi, Dear-Natura, Calcium, Magnesium, Zinc and Vitamin D‡ | アサヒ, ディアナチュラ, カルシウム・マグネシウム・亜鉛・ビタミンD | 30 | 31 | 31 | Tablet-multivitamins/minerals |
| Tablet, water-soluble vitamins |  |  | **600** |  |  |  |
|  | DHC, Vitamin C (hard capsule) (60-day supply) ‡ | DHC, ビタミンC（ハードカプセル）（60日分） | 210 | 211 | 210 | Tablet-single water-soluble vitamins |
|  | DHC, Vitamin B Mix (60-day supply) ‡ | DHC, ビタミンBミックス（60日分） | 110 | 113 | 113 | Tablet-single water-soluble vitamins |
|  | DHC, Sustained-release Vitamin C (60-day supply) ‡ | DHC, 持続型ビタミンC （60日分） | 90 | 87 | 87 | Tablet-single water-soluble vitamins |
|  | Asahi, Dear-Natura, Iron and Folic Acid (60-day supply) | アサヒ, ディアナチュラ　鉄・葉酸（60日分） | 60 | 60 | 60 | Tablet-folic acid and iron |
|  | Asahi, Dear-Natura Style, Folic Acid x Iron Calcium‡ | アサヒ, ディアナチュラスタイル　葉酸×鉄カルシウム | 70 | 69 | 69 | Tablet-folic acid and iron |
|  | DHC, Sustained-release Vitamin B Mix (60-day supply) ‡ | DHC, 持続型ビタミンBミックス（60日分） | 60 | 60 | 60 | Tablet-single water-soluble vitamins |
| Tablet, minerals |  |  | **500** |  |  |  |
|  | DHC, Zinc (60-day supply) ‡ | DHC, 亜鉛（60日分） | 280 | 279 | 279 | Tablet-single mineral |
|  | DHC, Heme Iron (60-day supply) ‡ | DHC, ヘム鉄（60日分） | 120 | 123 | 123 | Tablet-single mineral |
|  | DHC, Calcium and Magnesium (60-day supply) ‡ | DHC, カルシウムマグ（60日分） | 55 | 45 | 55 | Tablet-single mineral |
|  | DHC, Multimineral (60-day supply) ‡ | DHC, マルチミネラル（60日分） | 45 | 55 | 45 | Tablet-multivitamins/minerals |

*Those whose daily consumption was extremely high (n=2) were excluded because they were likely misreported.

† Products certified as “Designated Quasi-Drugs,” a category of products with mild, specific, and pre-approved, active ingredient-driven, efficacy and effects, aimed at prevention, hygiene, and wellness.

‡ Products labelled as “**Food with Nutrient Function Claims (*Eiyo-Kino-Shokuhin*).**” It refers to all food that is labeled with the nutrient function claims specified by the MHLW. The standards and specifications for indication of nutritional function have been so far established for 17 ingredients (12 vitamins and 5 minerals).

# **Table S2.** Characteristics of dietary supplement users (n=2,002) according to the dietary supplement form*.

|  | Form of dietary supplement | | | | |  |  |
| --- | --- | --- | --- | --- | --- | --- | --- |
|  | Liquid (N=301) | |  | Tablet (N=1701) | | P† | |
|  | N | (%) |  | N | (%) |  |  |
| Consumption of the dietary supplement compared to the manufacturer-recommended dose | | |  |  |  | <.0001 | |
| Below the recommended dose | 10 | (3.3) |  | 1002 | (59.4) |  | |
| Equal to the recommended dose | 277 | (92.0) |  | 342 | (20.3) |  | |
| Above the recommended dose | 14 | (4.7) |  | 357 | (21.1) |  | |
| Age (year) |  |  |  |  |  |  | |
| 18–34 years | 38 | (12.6) |  | 383 | (22.7) | 0.0004 | |
| 34–49 years | 149 | (49.5) |  | 807 | (47.8) |  | |
| 50–64 years | 106 | (35.2) |  | 461 | (27.3) |  | |
| ≥65 years | 8 | (2.7) |  | 50 | (3.0) |  | |
| Weight status |  |  |  |  |  | 0.004 | |
| Underweight (BMI: <18.5 kg/m^2^) | 30 | (10.0) |  | 268 | (15.9) |  | |
| Normal (BMI: 18.5–24.9 kg/m^2^) | 184 | (61.1) |  | 1008 | (59.7) |  | |
| Overweight or obesity (≥25 kg/m^2^) | 55 | (18.3) |  | 210 | (12.4) |  | |
| Missing | 32 | (10.6) |  | 215 | (12.7) |  | |
| Sex |  |  |  |  |  | <.0001 | |
| Mele | 104 | (34.6) |  | 384 | (22.7) |  | |
| Female | 197 | (65.4) |  | 1317 | (78.0) |  | |
| Educational level |  |  |  |  |  | 0.17 | |
| Junior or senior high school | 94 | (31.2) |  | 576 | (34.1) |  | |
| 2-year college or technical school | 89 | (29.6) |  | 499 | (29.6) |  | |
| University or higher | 112 | (37.2) |  | 557 | (33.0) |  | |
| Others or Refusal to answer | 6 | (2.0) |  | 69 | (4.1) |  | |
| Employment status |  |  |  |  |  | 0.004 | |
| Unemployed | 55 | (18.3) |  | 441 | (26.1) |  | |
| Student | 1 | (0.3) |  | 26 | (1.5) |  | |
| Part-time job | 79 | (26.2) |  | 454 | (26.9) |  | |
| Full-time job | 166 | (55.1) |  | 780 | (46.2) |  | |
| Medical history |  |  |  |  |  | 0.74 | |
| No | 186 | (61.8) |  | 1090 | (64.6) |  | |
| Yes | 100 | (33.2) |  | 529 | (31.3) |  | |
| Refusal to answer | 15 | (5.0) |  | 82 | (4.9) |  | |
| Smoking status |  |  |  |  |  | 0.12 | |
| Never | 210 | (69.8) |  | 1148 | (68.0) |  | |
| Past | 42 | (14.0) |  | 327 | (19.4) |  | |
| Current (< 20 cigarettes a day) | 40 | (13.3) |  | 179 | (10.6) |  | |
| Current (≥ 20 cigarettes a day) | 9 | (3.0) |  | 47 | (2.8) |  | |
| Drinking habit |  |  |  |  |  | 0.09 | |
| Not at all | 81 | (26.9) |  | 566 | (33.5) |  | |
| Hardly | 72 | (23.9) |  | 406 | (24.1) |  | |
| Sometimes | 109 | (36.2) |  | 507 | (30.0) |  | |
| Every day | 39 | (13.0) |  | 222 | (13.2) |  | |
| Pregnant (N, % of females) |  |  |  |  |  | 0.04 | |
| Yes | 1 | (0.5) |  | 41 | (3.1) |  | |
| No or refusal to answer | 196 | (99.5) |  | 1276 | (96.9) |  | |
| Breastfeeding (N, % of females) |  |  |  |  |  | 0.91 | |
| Yes | 6 | (3.0) |  | 42 | (3.2) |  | |
| No or refusal to answer | 191 | (97.0) |  | 1275 | (96.8) |  | |
| Duration of the dietary supplement use |  |  |  |  |  | <.0001 | |
| <3 months | 94 | (31.2) |  | 390 | (23.1) |  | |
| 3 months–<6 months | 47 | (15.6) |  | 241 | (14.3) |  | |
| 6 months–<1 year | 67 | (22.3) |  | 308 | (18.2) |  | |
| ≥1 year | 93 | (30.9) |  | 762 | (45.1) |  | |
| Checking the manufacture-recommended dose before use: "Before you started using the product, did you check the recommended dose of the product?" |  |  |  |  |  | <.0001 | |
| Yes | 98 | (32.6) |  | 1588 | (94.1) |  | |
| No | 203 | (67.4) |  | 113 | (6.7) |  | |
| Self-perception of their DS consumption: "Compared to the recommended dose, your consumption is…." |  |  |  |  |  | <.0001 | |
| More than the recommended dose | 6 | (2.0) |  | 76 | (4.5) |  | |
| Same as the recommended dose | 110 | (36.5) |  | 1158 | (68.6) |  | |
| Less than the recommended dose | 17 | (5.6) |  | 223 | (13.2) |  | |
| Unsure | 154 | (51.2) |  | 233 | (13.8) |  | |
| No recommended dose provided | 14 | (4.7) |  | 11 | (0.7) |  | |
| Food literacy score‡ |  |  |  |  |  |  | |
| Less than median score | 172 | (57.1) |  | 848 | (50.2) | 0.02 | |
| Same as the median score or more | 129 | (42.9) |  | 853 | (50.5) |  | |

*Participants were panellists who bought one of the 25 selected dietary supplements in the previous 3 months and used it within the past month or regularly. Information was collected from one of the 25 dietary supplements used by each participant.

† P values by X^2^ test for categorical variables. Statistical significance was set at P<0.05.

‡ The median score among participants was 3.17, with possible scores ranging from 1 to 5.

# **Table S3.** Number of participants and daily consumption of dietary supplements among 2,002 dietary supplement users*.

|  |  |  | Within-manufacture-recommended-dose (n=1631) | | | | | | | | |  | Exceeding-manufacture-  recommended-dose (n=371) § | | | |
| --- | --- | --- | --- | --- | --- | --- | --- | --- | --- | --- | --- | --- | --- | --- | --- | --- |
|  | Number of products | Analysed participants | Below the-manufacture-  recommended dose (n=619) † | | | |  | Equal to the -manufacture-  recommended dose (n=1012) ‡ | | | |  |  |  |  |  |
|  |  |  | N | (%) | Daily consumption compared to recommended dose (%)\|\| | |  | N | (%) | Daily consumption compared to recommended dose (%)\|\| | |  | N | (%) | Daily consumption compared to recommended dose (%)\|\| | |
|  |  |  |  |  | Mean | SD |  |  |  | Mean | SD |  |  |  | Mean | SD |
| Liquid | 2 | 299 | 277 | (92.0) | 12.3 | 15.4 |  | 10 | (3.3) | 100 | 0 |  | 14 | (4.7) | 490 | 355 |
| Tablet | 23 | 1688 | 342 | (20.1) | 45.5 | 20.6 |  | 1002 | (58.9) | 100 | 0 |  | 357 | (21.0) | 463 | 419 |
| *Multivitamins/multimineral* | *8* | *482* | *92* | *(18.9)* | *48.5* | *23.2* |  | *302* | *(62.1)* | *100* | *0* |  | *92* | *(18.9)* | *472* | *363* |
| *Folic acid and iron* | *2* | *129* | *23* | *(17.8)* | *47.5* | *18.6* |  | *85* | *(65.9)* | *100* | *0* |  | *21* | *(16.3)* | *543* | *146* |
| *Single fat-soluble vitamins* | *6* | *167* | *23* | *(13.6)* | *43.9* | *20.6* |  | *109* | *(64.5)* | *100* | *0* |  | *37* | *(21.9)* | *488* | *393* |
| *Single water-soluble vitamins* | *4* | *466* | *107* | *(22.8)* | *43.7* | *19.0* |  | *240* | *(51.1)* | *100* | *0* |  | *123* | *(26.2)* | *394* | *269* |
| *Single minerals* | *3* | *444* | *97* | *(21.7)* | *44.4* | *20.0* |  | *266* | *(59.5)* | *100* | *0* |  | *84* | *(18.8)* | *525* | *645* |

*Participants were panellists who had a purchase history of one of the 25 selected dietary supplements in the last 3 months and used it within the past month or regularly. Information was collected from one of the 25 dietary supplements used by each participant.

†Those whose daily intake of the dietary supplement was equal to the manufacturer-recommended dose

‡Those whose daily intake of the dietary supplement was below the manufacturer-recommended dose

§Those whose intake exceeded the manufacturer-recommended dose.

||Calculated as dietary supplement consumption (that is, number of tablets or ml of liquid per day) per recommended dose per day of the product.

# **Table S4.** Characteristics of dietary supplement users (n=1821) according to dietary supplement consumption compared with the recommended dose*.

|  | All (n=1821) | |  | Within-manufacture-recommended-dose (n=1631)† | |  | Exceeding-manufacture-recommended-dose (n=190)‡ | | P§ |
| --- | --- | --- | --- | --- | --- | --- | --- | --- | --- |
|  | N | (%) |  | N | (%) |  | N | (%) |  |
| Age |  |  |  |  |  |  |  |  |  |
| 18–34 years | 380 | (20.9) |  | 337 | (20.7) |  | 43 | (22.6) | 0.14 |
| 34–49 years | 880 | (48.3) |  | 803 | (49.2) |  | 77 | (40.5) |  |
| 50–64 years | 507 | (27.8) |  | 444 | (27.2) |  | 63 | (33.2) |  |
| ≥65 years | 54 | (3.0) |  | 47 | (2.9) |  | 7 | (3.7) |  |
| Weight status |  |  |  |  |  |  |  |  | 0.85 |
| Underweight (BMI: <18.5 kg/m^2^) | 280 | (15.4) |  | 253 | (15.5) |  | 27 | (14.2) |  |
| Normal (BMI: 18.5–24.9 kg/m^2^) | 1079 | (59.3) |  | 961 | (58.9) |  | 118 | (62.1) |  |
| Overweight or obesity (≥25 kg/m^2^) | 237 | (13.0) |  | 213 | (13.1) |  | 24 | (12.6) |  |
| Missing | 225 | (12.4) |  | 204 | (12.5) |  | 21 | (11.1) |  |
| Sex |  |  |  |  |  |  |  |  | 0.36 |
| Mele | 431 | (23.7) |  | 381 | (23.4) |  | 50 | (26.3) |  |
| Female | 1390 | (76.3) |  | 1250 | (76.6) |  | 140 | (73.7) |  |
| Educational level |  |  |  |  |  |  |  |  | 0.14 |
| Junior or senior high school | 596 | (32.7) |  | 539 | (33.0) |  | 57 | (30.0) |  |
| 2-year college or technical school | 535 | (29.4) |  | 472 | (28.9) |  | 63 | (33.2) |  |
| University or higher | 619 | (34.0) |  | 561 | (34.4) |  | 58 | (30.5) |  |
| Others or refusal to answer | 71 | (3.9) |  | 59 | (3.6) |  | 12 | (6.3) |  |
| Employment status |  |  |  |  |  |  |  |  | 0.005 |
| Unemployed | 453 | (24.9) |  | 424 | (26.0) |  | 29 | (15.3) |  |
| Student | 23 | (1.3) |  | 20 | (1.2) |  | 3 | (1.6) |  |
| Part-time job | 493 | (27.1) |  | 427 | (26.2) |  | 66 | (34.7) |  |
| Full-time job | 852 | (46.8) |  | 760 | (46.6) |  | 92 | (48.4) |  |
| Medical history |  |  |  |  |  |  |  |  | 0.77 |
| No | 1158 | (63.6) |  | 1034 | (63.4) |  | 124 | (65.3) |  |
| Yes | 579 | (31.8) |  | 520 | (31.9) |  | 59 | (31.1) |  |
| Refusal to answer | 84 | (4.6) |  | 77 | (4.7) |  | 7 | (3.7) |  |
| Smoking status |  |  |  |  |  |  |  |  | 0.18 |
| Never | 1245 | (68.4) |  | 1125 | (69.0) |  | 120 | (63.2) |  |
| Past | 332 | (18.2) |  | 297 | (18.2) |  | 35 | (18.4) |  |
| Current (< 20 cigarettes a day) | 196 | (10.8) |  | 168 | (10.3) |  | 28 | (14.7) |  |
| Current (≥20 cigarettes a day) | 48 | (2.6) |  | 41 | (2.5) |  | 7 | (3.7) |  |
| Drinking habit |  |  |  |  |  |  |  |  | 0.006 |
| Not at all | 588 | (32.3) |  | 524 | (32.1) |  | 64 | (33.7) |  |
| Hardly | 438 | (24.1) |  | 411 | (25.2) |  | 27 | (14.2) |  |
| Sometimes | 570 | (31.3) |  | 501 | (30.7) |  | 69 | (36.3) |  |
| Every day | 225 | (12.4) |  | 195 | (12.0) |  | 30 | (15.8) |  |
| Pregnant (N, % of females) |  |  |  |  |  |  |  |  | 0.34 |
| Yes | 37 | (2.7) |  | 35 | (2.8) |  | 2 | (1.4) |  |
| No or refusal to answer | 1353 | (97.3) |  | 1215 | (97.2) |  | 138 | (98.6) |  |
| Breastfeeding (N, % of females) |  |  |  |  |  |  |  |  | 0.99 |
| Yes | 40 | (2.9) |  | 36 | (2.9) |  | 4 | (2.9) |  |
| No or refusal to answer | 1350 | (97.1) |  | 1214 | (97.1) |  | 136 | (97.1) |  |
| Form of dietary supplement |  |  |  |  |  |  |  |  | <.0001 |
| Liquid | 296 | (16.3) |  | 287 | (17.6) |  | 9 | (4.7) |  |
| Tablet | 1526 | (83.8) |  | 1345 | (82.5) |  | 181 | (95.3) |  |
| Featured nutrients of the dietary supplement (tablet form only) \|\| | |  |  |  |  |  |  |  | <.0001 |
| Multivitamins/multimineral | 440 | (24.2) |  | 394 | (24.2) |  | 46 | (24.2) |  |
| Folic acid and iron | 111 | (6.1) |  | 108 | (6.6) |  | 3 | (1.6) |  |
| Single fat-soluble vitamins | 150 | (8.2) |  | 132 | (8.1) |  | 18 | (9.5) |  |
| Single water soluble vitamins | 425 | (23.3) |  | 347 | (21.3) |  | 78 | (41.1) |  |
| Single minerals | 399 | (21.9) |  | 363 | (22.3) |  | 36 | (18.9) |  |
| Duration of the dietary supplement use |  |  |  |  |  |  |  |  | 0.058 |
| <3 months | 458 | (25.2) |  | 420 | (25.8) |  | 38 | (20.0) |  |
| 3 months–<6 months | 265 | (14.6) |  | 245 | (15.0) |  | 20 | (10.5) |  |
| 6 months–<1 year | 344 | (18.9) |  | 303 | (18.6) |  | 41 | (21.6) |  |
| ≥1 year | 755 | (41.5) |  | 664 | (40.7) |  | 91 | (47.9) |  |
| Checking the manufacture-recommended dose before use: "Before you started using the product, did you check the recommended dose of the product?" |  |  |  |  |  |  |  |  | 0.01 |
| Yes | 1522 | (83.6) |  | 1351 | (82.8) |  | 171 | (90.0) |  |
| No | 299 | (16.4) |  | 280 | (17.2) |  | 19 | (10.0) |  |
| Self-perception of their DS consumption: "Compared to the recommended dose, your consumption is…." |  |  |  |  |  |  |  |  | <.0001 |
| More than the recommended dose | 64 | (3.5) |  | 25 | (1.5) |  | 39 | (20.5) |  |
| Same as the recommended dose | 1147 | (63.0) |  | 1056 | (64.7) |  | 91 | (47.9) |  |
| Less than the recommended dose | 227 | (12.5) |  | 200 | (12.3) |  | 27 | (14.2) |  |
| Unsure | 358 | (19.7) |  | 326 | (20.0) |  | 32 | (16.8) |  |
| No recommended dose provided | 25 | (1.4) |  | 24 | (1.5) |  | 1 | (0.5) |  |
| Food literacy score⁋ |  |  |  |  |  |  |  |  |  |
| Less than median score | 925 | (50.8) |  | 837 | (51.3) |  | 88 | (46.3) | 0.19 |
| Same as median score or more | 896 | (49.2) |  | 794 | (48.7) |  | 102 | (53.7) |  |

*Participants were panellists who bought one of the 25 selected dietary supplements in the previous 3 months and used it within the past month or regularly. Information was collected from one of the 25 dietary supplements used by each participant. Overall, 1,821 participants were analysed after excluding those who consumed DS five times or more than the manufacturer-recommended dose (n=183).

†Those whose daily intake of the dietary supplement was equal to or below the manufacturer-recommended dose.

‡Those whose intake exceeded the manufacturer-recommended dose.

§P values by X^2^ test for categorical variables. Statistical significance was set at P<0.05.

||"Multivitamins" contained three or more vitamins or minerals. "Folic acid and iron" contained folic acid and iron as the primary nutrients. "Single fat-soluble vitamins" contained <3 fat-soluble vitamins as the primary nutrients. "Single water-soluble vitamins" contained <3 water-soluble vitamins as the primary nutrients. "Single mineral" contained <3 minerals as the primary nutrients.

⁋ The median score among the participants was 3.18, with possible scores ranging from 1–5.

# **Table S5.** Factors associated with dietary supplement consumption exceeding the manufacturer-recommended doses among 1,821 dietary supplement users.

|  | Crude model† | |  |  | Adjusted model‡ | |  |
| --- | --- | --- | --- | --- | --- | --- | --- |
|  | OR | (95%CI) | p-for trend |  | OR | (95%CI) | p-for trend |
| Age |  |  | 0.26 |  |  |  | 0.12 |
| 18–34 years | 1.00 | (Ref) |  |  | 1.00 | (Ref) |  |
| 34–49 years | 1.33 | (0.90, 1.97) |  |  | 1.25 | (0.84, 1.87) |  |
| 50–64 years | 1.48 | (1.04, 2.11) |  |  | 1.55 | (1.07, 2.24) |  |
| ≥65 years | 1.55 | (0.68, 3.55) |  |  | 1.76 | (0.75, 4.17) |  |
| Weight status§ |  |  |  |  |  |  |  |
| Underweight (BMI: <18.5 kg/m^2^) | 0.87 | (0.56, 1.35) |  |  | 0.87 | (0.56, 1.37) |  |
| Normal (BMI: 18.5–24.9 kg/m^2^) | 1.00 | (Ref) |  |  | 1.00 | (Ref) |  |
| Overweight or obesity (≥25 kg/m^2^) | 0.92 | (0.58, 1.46) |  |  | 0.92 | (0.57, 1.50) |  |
| Missing | 0.84 | (0.52, 1.37) |  |  | 0.85 | (0.52, 1.40) |  |
| Sex |  |  |  |  |  |  |  |
| Mele | 1.00 | (Ref) |  |  | 1.00 | (Ref) |  |
| Female | 0.85 | (0.61, 1.20) |  |  | 0.95 | (0.65, 1.37) |  |
| Educational level |  |  | 0.51 |  |  |  | 0.42 |
| Junior or senior high school | 1.00 | (Ref) |  |  | 1.00 | (Ref) |  |
| 2-year college or technical school | 1.26 | (0.86, 1.84) |  |  | 1.33 | (0.90, 1.96) |  |
| University or higher | 0.98 | (0.67, 1.44) |  |  | 0.98 | (0.66, 1.46) |  |
| Others or Refusal to answer | 1.92 | (0.98, 3.79) |  |  | 2.14 | (1.06, 4.34) |  |
| Employment status |  |  | 0.014 |  |  |  | 0.009 |
| Unemployed | 1.00 | (Ref) |  |  | 1.00 | (Ref) |  |
| Student | 2.19 | (0.62, 7.81) |  |  | 2.33 | (0.62, 8.71) |  |
| Part-time job | 2.26 | (1.43, 3.57) |  |  | 2.43 | (1.53, 3.85) |  |
| Full-time job | 1.77 | (1.15, 2.73) |  |  | 1.83 | (1.17, 2.87) |  |
| Medical history |  |  |  |  |  |  |  |
| Yes | 0.95 | (0.68, 1.31) |  |  | 0.93 | (0.66, 1.32) |  |
| No | 1.00 | (Ref) |  |  | 1.00 | (Ref) |  |
| Refusal to answer | 0.76 | (0.34, 1.68) |  |  | 0.79 | (0.35, 1.78) |  |
| Smoking status |  |  | 0.038 |  |  |  | 0.14 |
| Never | 1.00 | (Ref) |  |  | 1.00 | (Ref) |  |
| Past | 1.11 | (0.74, 1.64) |  |  | 0.97 | (0.63, 1.48) |  |
| Current (< 20 cigarettes a day) | 1.56 | (1.00, 2.43) |  |  | 1.51 | (0.95, 2.42) |  |
| Current (≥20 cigarettes a day) | 1.60 | (0.70, 3.65) |  |  | 1.32 | (0.56, 3.10) |  |
| Drinking habit |  |  | 0.14 |  |  |  | 0.38 |
| Not at all | 1.00 | (Ref) |  |  | 1.00 | (Ref) |  |
| Hardly | 0.54 | (0.34, 0.86) |  |  | 0.53 | (0.33, 0.85) |  |
| Sometimes | 1.13 | (0.79, 1.62) |  |  | 1.12 | (0.77, 1.62) |  |
| Every day | 1.26 | (0.79, 2.00) |  |  | 1.06 | (0.65, 1.74) |  |
| Form of dietary supplement |  |  |  |  |  |  |  |
| Liquid type | 1.00 | (Ref) |  |  | 1.00 | (Ref) |  |
| Tablet type | 4.30 | (2.17, 8.49) |  |  | 4.72 | (2.38, 9.36) |  |
| Featured nutrients of the dietary supplement (tablet type only) §\|\| | | |  |  |  |  |  |
| Multivitamins/multimineral | 1.00 | (Ref) |  |  | 1.00 | (Ref) |  |
| Folic acid and iron | 0.24 | (0.07, 0.78) |  |  | 0.26 | (0.08, 0.87) |  |
| Single fat-soluble vitamins | 1.17 | (0.65, 2.09) |  |  | 1.15 | (0.64, 2.06) |  |
| Single water-soluble vitamins | 1.93 | (1.30, 2.85) |  |  | 1.91 | (1.28, 2.84) |  |
| Single minerals | 0.85 | (0.54, 1.34) |  |  | 0.82 | (0.52, 1.31) |  |
| Duration of the dietary supplement use |  |  | 0.012 |  |  |  | 0.045 |
| <3 months | 1.00 | (Ref) |  |  | 1.00 | (Ref) |  |
| 3 months–<6 months | 0.91 | (0.52, 1.59) |  |  | 0.85 | (0.48, 1.50) |  |
| 6 months–<1 year | 1.50 | (0.94, 2.38) |  |  | 1.49 | (0.93, 2.38) |  |
| ≥1 year | 1.52 | (1.02, 2.26) |  |  | 1.36 | (0.91, 2.04) |  |
| Checking the manufacture-recommended dose before use: "Before you started using the product, did you check the recommended dose of the product?" |  |  |  |  |  |  |  |
| Yes | 1.22 | (0.90, 1.65) |  |  | 1.08 | (0.61, 1.90) |  |
| No | 1.00 | (Ref) |  |  | 1.00 | (Ref) |  |
| Self-perception of their DS consumption: "Compared to the recommended dose, your consumption is…." |  |  |  |  |  |  |  |
| More than the recommended dose | 18.10 | (10.49, 31.24) |  |  | 18.70 | (10.64, 32.87) |  |
| Same as the recommended dose | 1.00 | (Ref) |  |  | 1.00 | (Ref) |  |
| Less than the recommended dose | 1.57 | (0.99, 2.47) |  |  | 1.55 | (0.98, 2.46) |  |
| Unsure | 1.14 | (0.75, 1.74) |  |  | 1.62 | (1.04, 2.53) |  |
| No recommended dose provided | 0.48 | (0.07, 3.62) |  |  | 0.80 | (0.10, 6.14) |  |
| Food literacy score¶ |  |  |  |  |  |  |  |
| Less than median score | 1.00 | (Ref) |  |  | 1.00 | (Ref) |  |
| Same as median score or more | 1.22 | (0.90, 1.65) |  |  | 1.16 | (0.85, 1.57) |  |

OR, Odds ratio; AOR, adjusted odds ratio.

*Participants were panellists who bought one of the 25 selected dietary supplements in the previous 3 months and used it within the past month or regularly. Information was collected from one of the 25 dietary supplements used by each participant. Overall, 1,821 participants were analysed after excluding those who consumed DS five times or more than the manufacturer-recommended dose (n=183).

†Derived from univariable logistic regression models.

‡Derived from multivariable logistic regression models mutually adjusted for sex, age, weight status, educational level, employment status, medical history, smoking status, drinking habit, and form of dietary supplement; excluded for main variables.

§"Multivitamins/multimineral" contained three or more vitamins or minerals. "Folic acid and iron" contained folic acid and iron as the primary nutrients. "Single fat-soluble vitamins" contained <3 fat-soluble vitamins as the primary nutrients. "Single water-soluble vitamins" contained <3 water-soluble vitamins as the primary nutrients. "Single mineral" contained <3 minerals as the primary nutrients.

||Users of the liquid-form DS were excluded from the analysis. The form of the dietary supplement was not adjusted.

¶ The median score among participants was 3.18, with possible scores ranging from 1 to 5.

# **Table S6.** Nutrient intake with defined UL among dietary supplement users (n=1,821).

| Nutrients | UL† | All (n=1821) | |  | Within-manufacture-recommended-dose (n=1631)‡ | | | |  | Exceeding-manufacture-recommended-dose (n=190)§ | | | |
| --- | --- | --- | --- | --- | --- | --- | --- | --- | --- | --- | --- | --- | --- |
|  |  | N\|\| | Exceed UL |  | N\|\| | Nutrient Intake | | Exceed UL |  | N\|\| | Nutrient Intake | | Exceed UL |
|  |  |  | N (%) |  |  | Median | 25th, 75th percentile | N (%) |  |  | Median | 25th, 75th percentile | N (%) |
| Vitamin A (μg) | 2700 | 359 | 5 (1.3) |  | 327 | 770 | 450, 770 | 0 (0.0) |  | 32 | 1540 | 982, 2355 | 5 (15.6) |
| Vitamin D (μg) | 100 | 582 | 0 (0.0) |  | 527 | 6.6 | 5.0, 10.0 | 0 (0.0) |  | 55 | 20.0 | 10.0, 28.6 | 0 (0.0) |
| Vitamin E (mg) | Men: 800  Women aged 18-29 years: 650 Women aged 30-74 years: 700 | 420 | 0 (0.0) |  | 381 | 6.3 | 6.3, 10.0 | 0 (0.0) |  | 39 | 18.9 | 12.6, 25.2 | 0 (0.0) |
| Niacin (mg)⁋ | Men, aged 18–29 years and 65-74 years: 80 Men, aged 30–64 years: 85 Women: 65 | 809 | 21 (2.4) |  | 746 | 13.0 | 2.1, 15.0 | 0 (0.0) |  | 63 | 54.0 | 30.0, 80.0 | 21 (33.3) |
| Vitamin B_6_ (mg) | Men, aged 18–29 years: 55 Men, aged 30–64 years: 60 Women: 45 | 871 | 17 (1.8) |  | 805 | 1.3 | 0.7, 3.2 | 0 (0.0) |  | 66 | 11.2 | 4.2, 60.0 | 17 (25.8) |
| Folic acid (μg) | Aged 18–29 years, ≥65 years: 900 Aged 30-64y: 1000 | 737 | 2 (0.2) |  | 667 | 200 | 100, 240 | 0 (0.0) |  | 70 | 400 | 300, 504 | 2 (2.9) |
| Calcium (mg) | 2500 | 249 | 3 (1.1) |  | 216 | 200 | 90, 360 | 0 (0.0) |  | 33 | 500 | 400, 1000 | 3 (9.1) |
| Magnesium (mg) | 350** | 187 | 12 (6.4) |  | 157 | 125 | 100, 250 | 0 (0) |  | 30 | 270 | 200, 500 | 12 (40.0) |
| Zinc (mg) | Men, aged 18–29years: 40 Men, aged 30–74years: 45 Women: 35 | 476 | 4 (0.9) |  | 348 | 15.0 | 6.0, 15.0 | 0 (0.0) |  | 41 | 18.0 | 12.0, 30.0 | 4 (9.8) |
| Copper (mg) | 7 | 389 | 0 (0.0) |  | 94 | 0.6 | 0.2, 0.6 | 0 (0.0) |  | 21 | 1.08 | 0.8, 1.2 | 0 (0.0) |
| Manganese (mg) | 11 | 115 | 0 (0.0) |  | 68 | 1.5 | 0.9, 1.5 | 0 (0.0) |  | 18 | 3.0 | 2.2, 4.3 | 0 (0.0) |
| Iodine (μg) | 3000 | 86 | 0 (0.0) |  | 68 | 45.0 | 25.6, 50.8 | 0 (0.0) |  | 18 | 95.8 | 67.7, 108 | 0 (0.0) |
| Selenium (μg) | Men, aged 18–29 years: 400 Men, aged 30–74 years: 450 Women: 350 | 86 | 0 (0.0) |  | 321 | 50.0 | 30.2, 50.0 | 0 (0.0) |  | 38 | 100 | 57.6, 100 | 0 (0.0) |
| Chromium (μg) | 500 | 359 | 0 (0.0) |  | 321 | 60.0 | 21.4, 60.0 | 0 (0.0) |  | 38 | 78.5 | 40.0, 120 | 0 (0.0) |
| Molybdenum (μg) | Men: 600 Women: 500 | 359 | 0 (0.0) |  | 68 | 9.0 | 5.2, 10.5 | 0 (0.0) |  | 18 | 19.5 | 14.0, 21.6 | 0 (0.0) |
| ≥1 nutrients above |  | 1550 | 44 (2.8) |  | 1416 | - | - | 0 (0.0) |  | 134 | - | - | 44 (32.8) |

UL: tolerable upper intake level

*Participants were panellists who had bought one of the 25 selected dietary supplements in the last 3 months and used it within the past month or regularly. Information was collected from one of the 25 dietary supplements used by each participant. Overall, 1,821 participants were analysed after excluding those who consumed DS five times or more than the manufacturer-recommended dose (n=183).

†UL is defined by the Dietary Reference Intake for Japan, 2025.

‡Those whose daily intake of the dietary supplement was equal to or below the manufacturer-recommended amount.

§Those whose intake exceeded the manufacturer-recommended amount.

||Number of participants taking nutrient-containing dietary supplements in the leftmost column.

⁋Amount of nicotinamide.

** ULs were defined for intakes derived from dietary supplements only.
